# Supplementary material for: Modulatory Effect of Guinep (Melicoccus bijugatus Jacq) Fruit Pulp Extract on Isoproterenol-Induced Myocardial Damage in Rats. Identification of Major Metabolites Using High Resolution UHPLC Q-Orbitrap Mass Spectrometry
Source: Molecules. 2019 Jan 10;24(2):235. doi: 10.3390/molecules24020235 (PMC6359499; doi:10.3390/molecules24020235)
Supplement: Supplementary file 1 [file molecules-24-00235-s001.pdf]

Supplementary material

Figure S1: Quadrupole Orbitrap full MS spectra and structures of all detected compounds, in fruits of *Melicoccus bijugatus.*

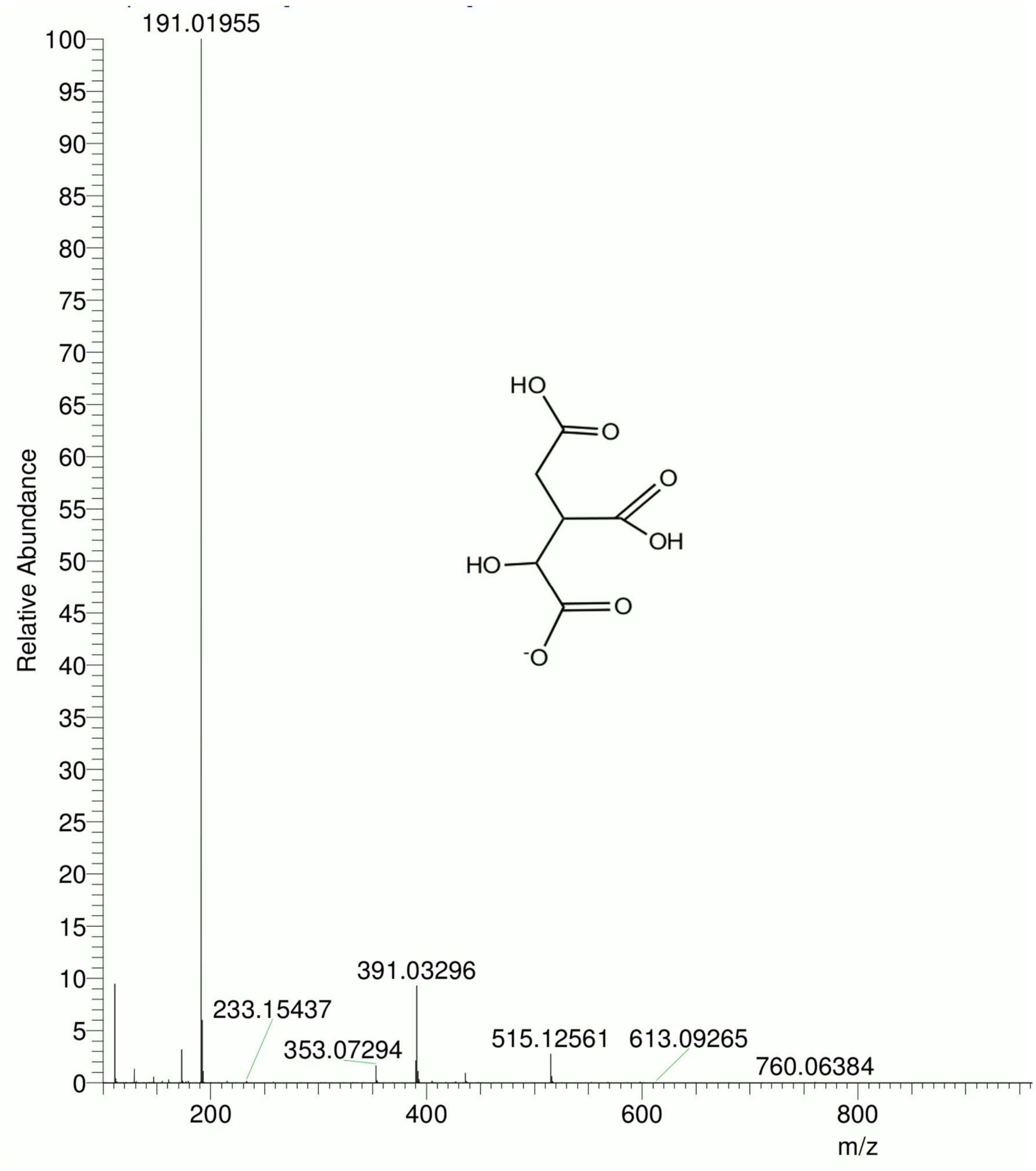

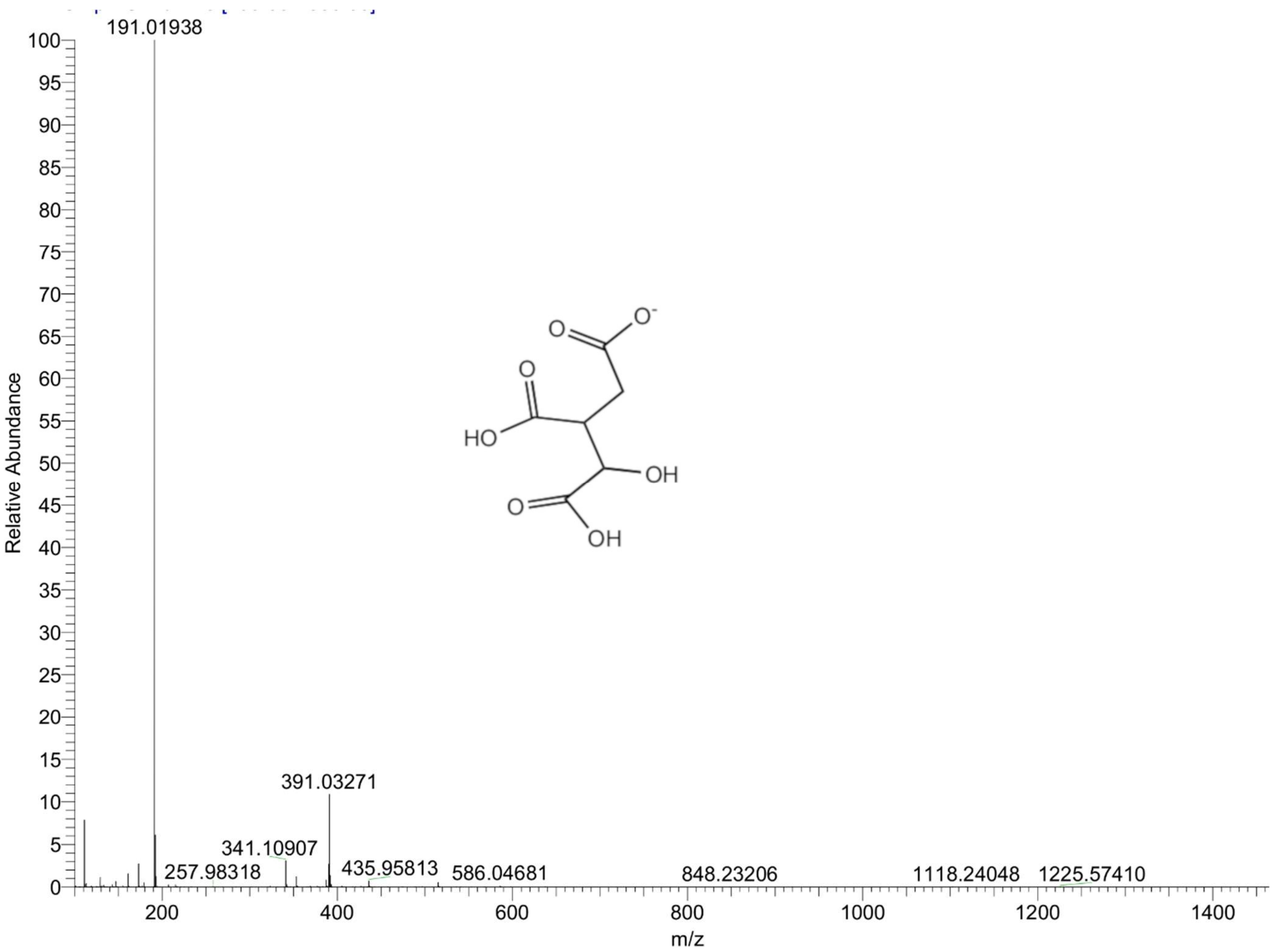
191.01938
391.03271
341.10907
257.98318
435.95813
586.04681
848.23206
1118.24048
1225.57410
Relative Abundance
m/z
0
5
10
15
20
25
30
35
40
45
50
55
60
65
70
75
80
85
90
95
100
200
400
600
800
1000
1200
1400
O
O⁻
O
HO
OH
O
OH

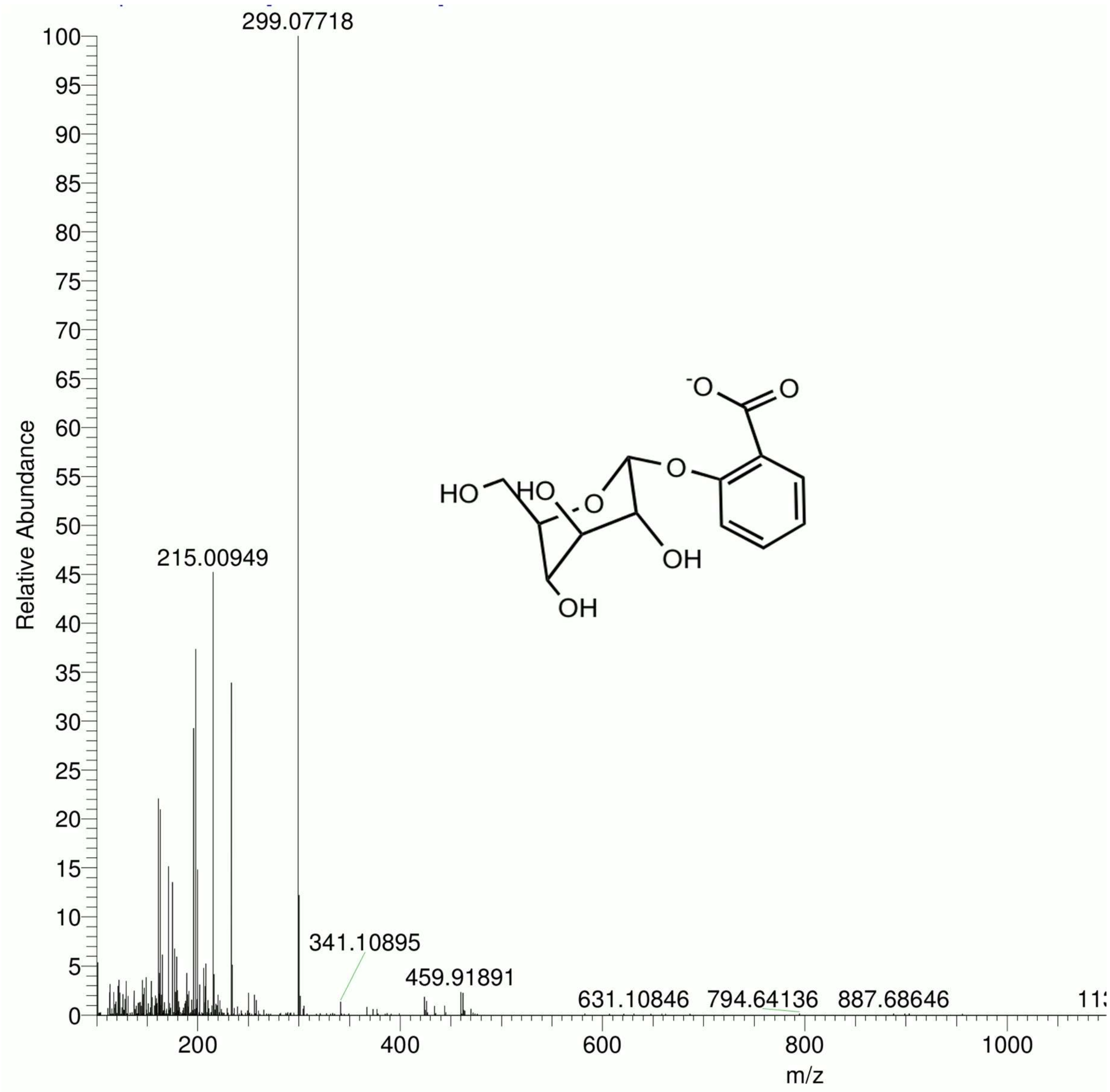

299.07718
215.00949
341.10895
459.91891
631.10846
794.64136
887.68646
Relative Abundance
m/z
HO
HO
O
O
OH
OH
-O
O

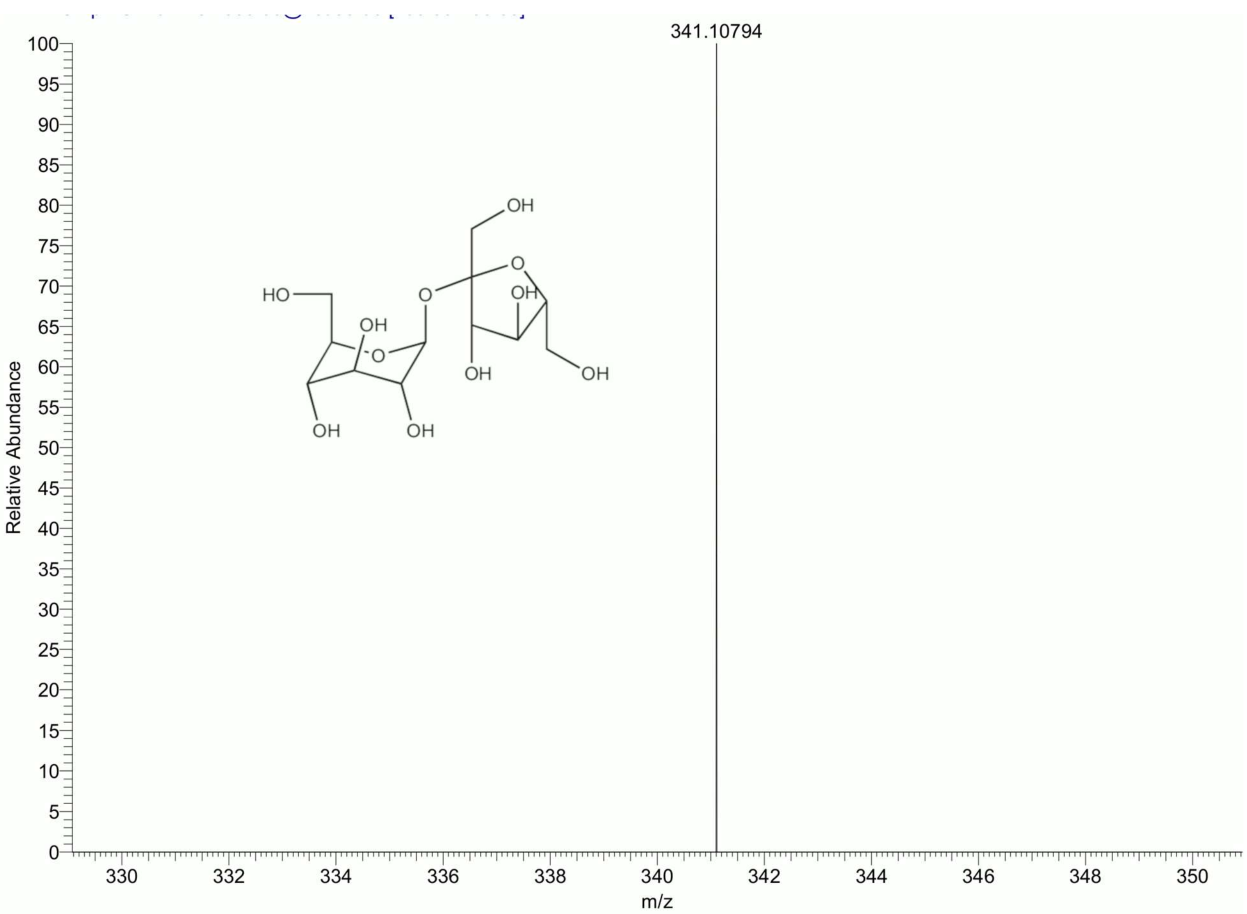

341.10794
Relative Abundance
m/z
100
95
90
85
80
75
70
65
60
55
50
45
40
35
30
25
20
15
10
5
0
330
332
334
336
338
340
342
344
346
348
350
HO
OH
O
OH
OH
OH
OH
OH
OH

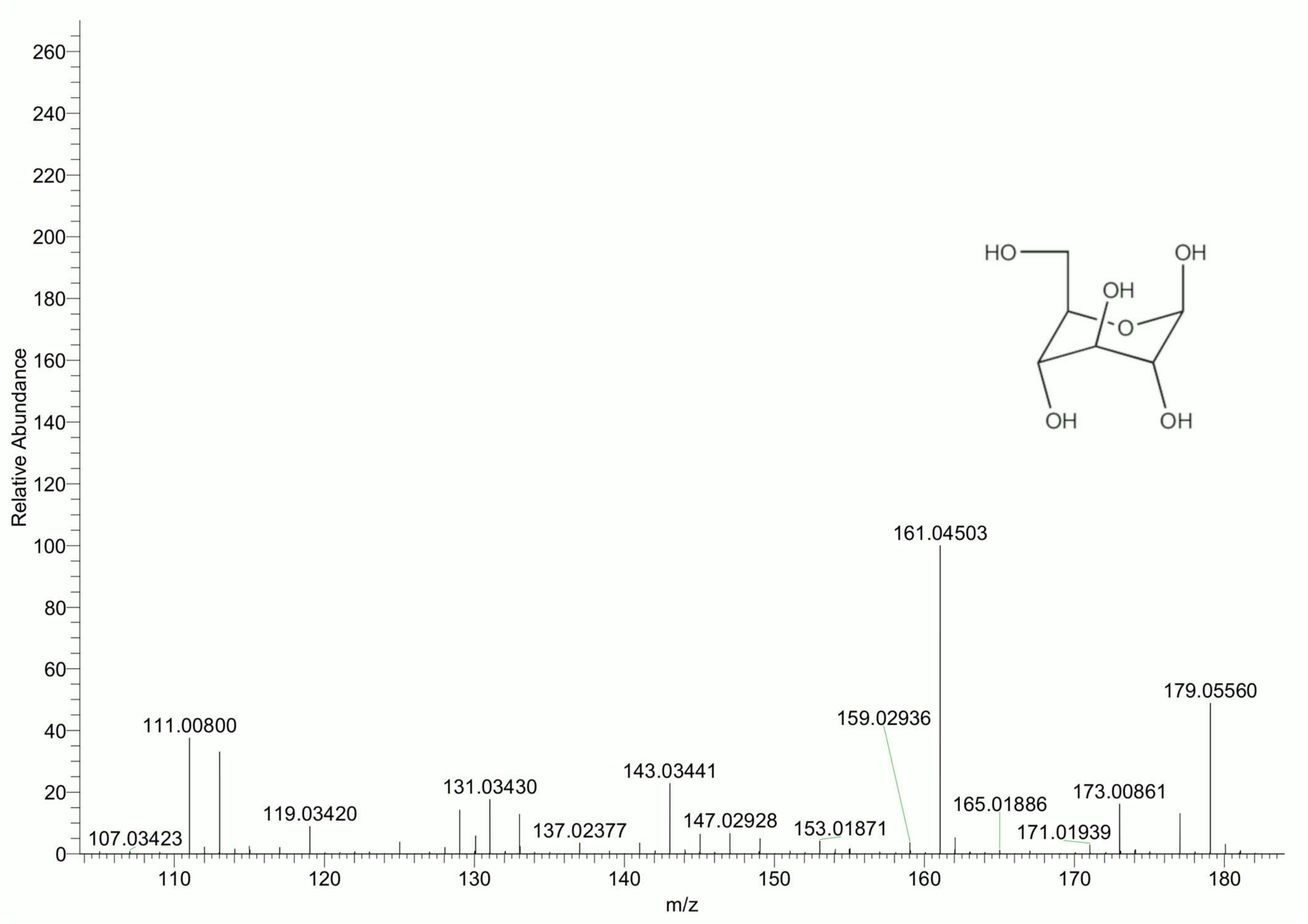
Relative Abundance
m/z
161.04503
179.05560
111.00800
159.02936
143.03441
131.03430
173.00861
165.01886
119.03420
147.02928
153.01871
137.02377
171.01939
107.03423
HO
OH
OH
O
OH
OH

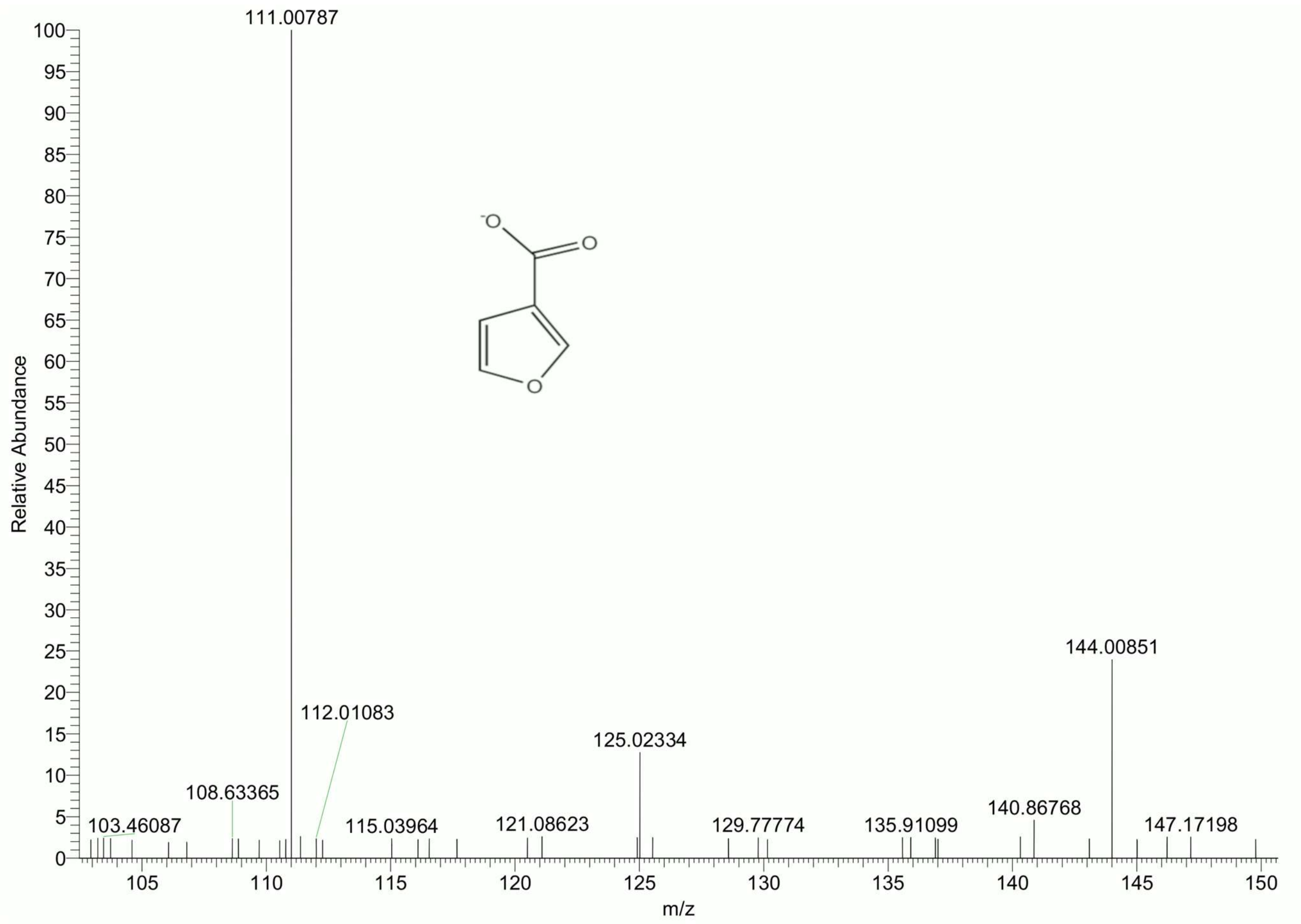

111.00787
144.00851
112.01083
125.02334
108.63365
103.46087
115.03964
121.08623
129.77774
135.91099
140.86768
147.17198
Relative Abundance
m/z
O
O
O

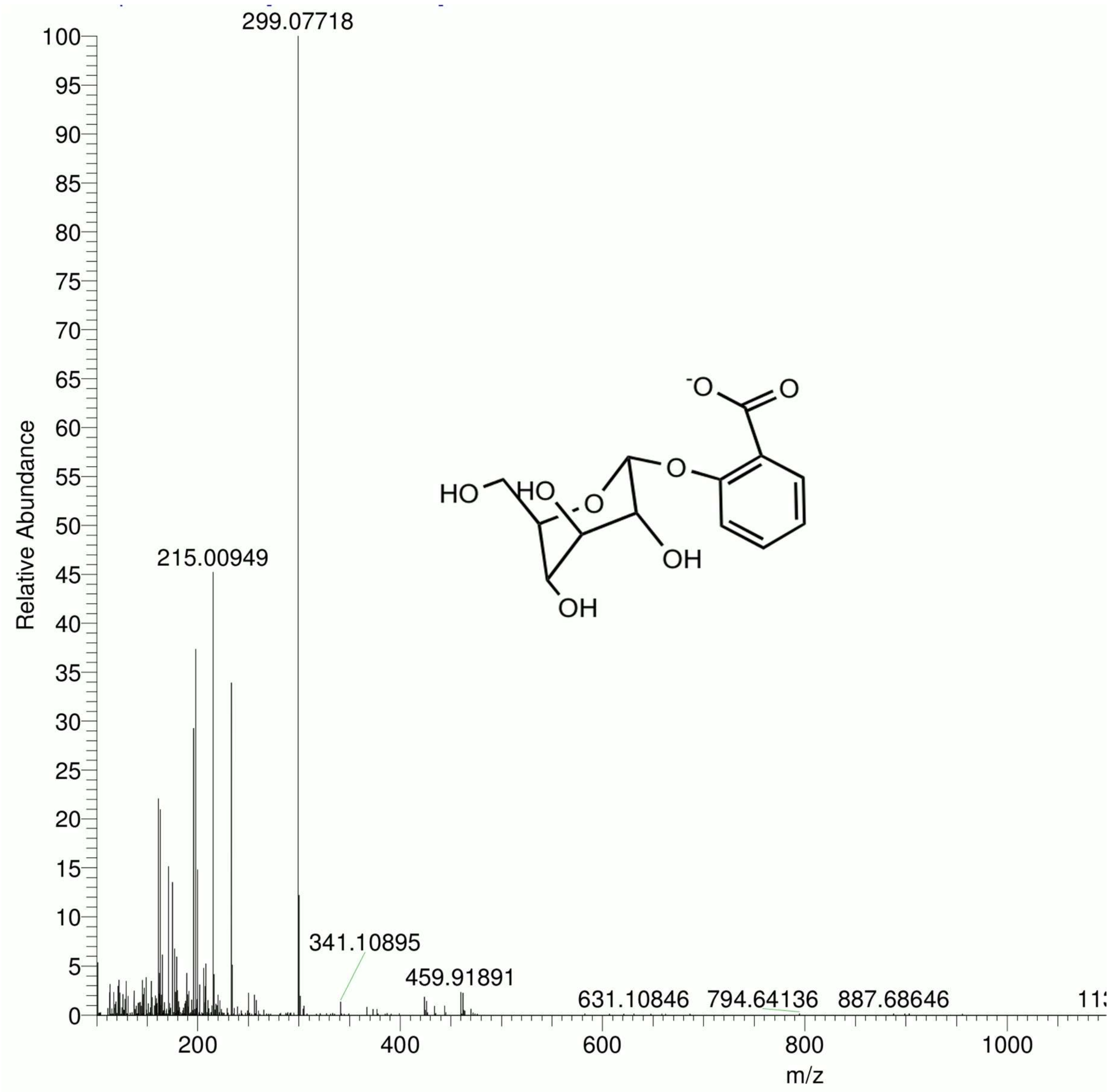

299.07718
215.00949
341.10895
459.91891
631.10846
794.64136
887.68646
Relative Abundance
m/z
HO
HO
O
O
OH
OH
O
-O

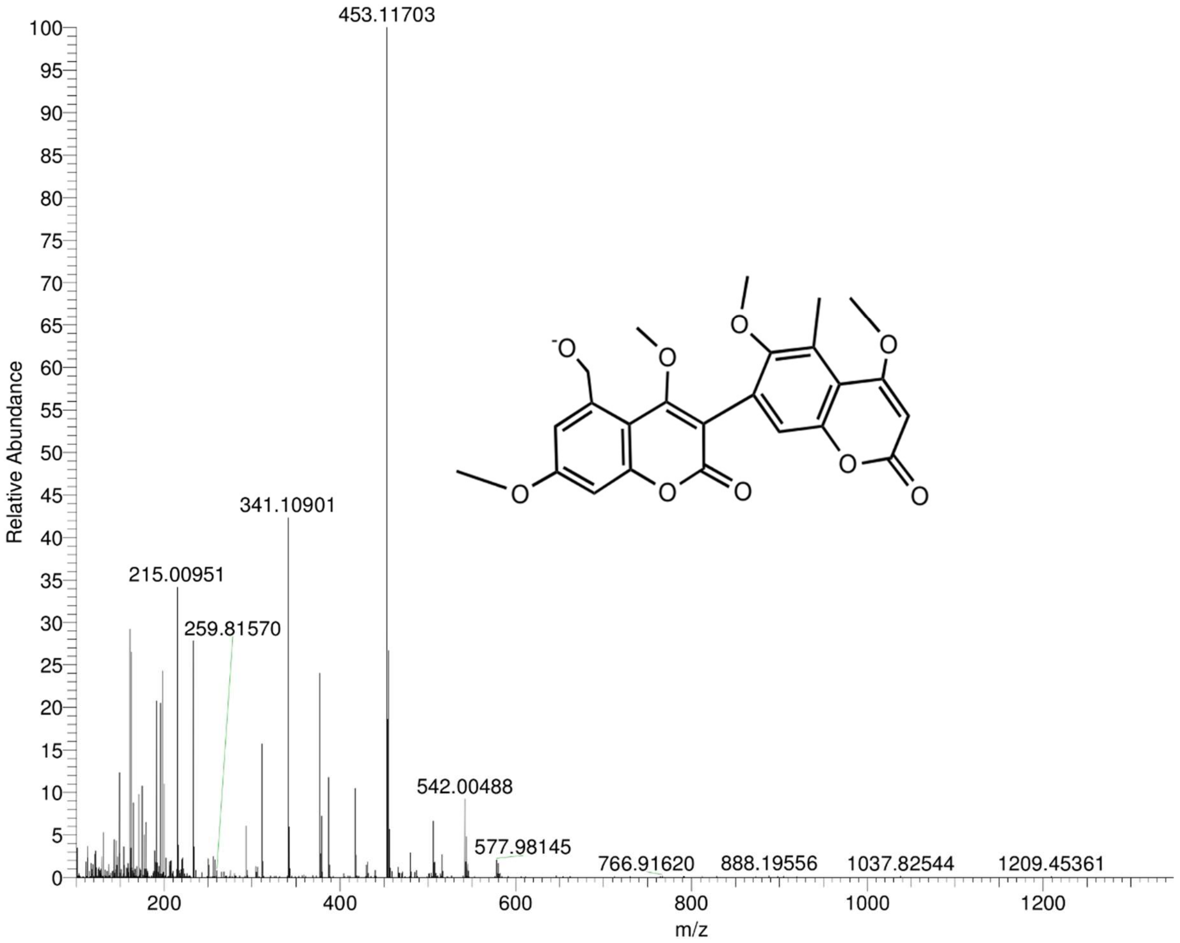

453.11703
341.10901
215.00951
259.81570
542.00488
577.98145
766.91620
888.19556
1037.82544
1209.45361
Relative Abundance
m/z

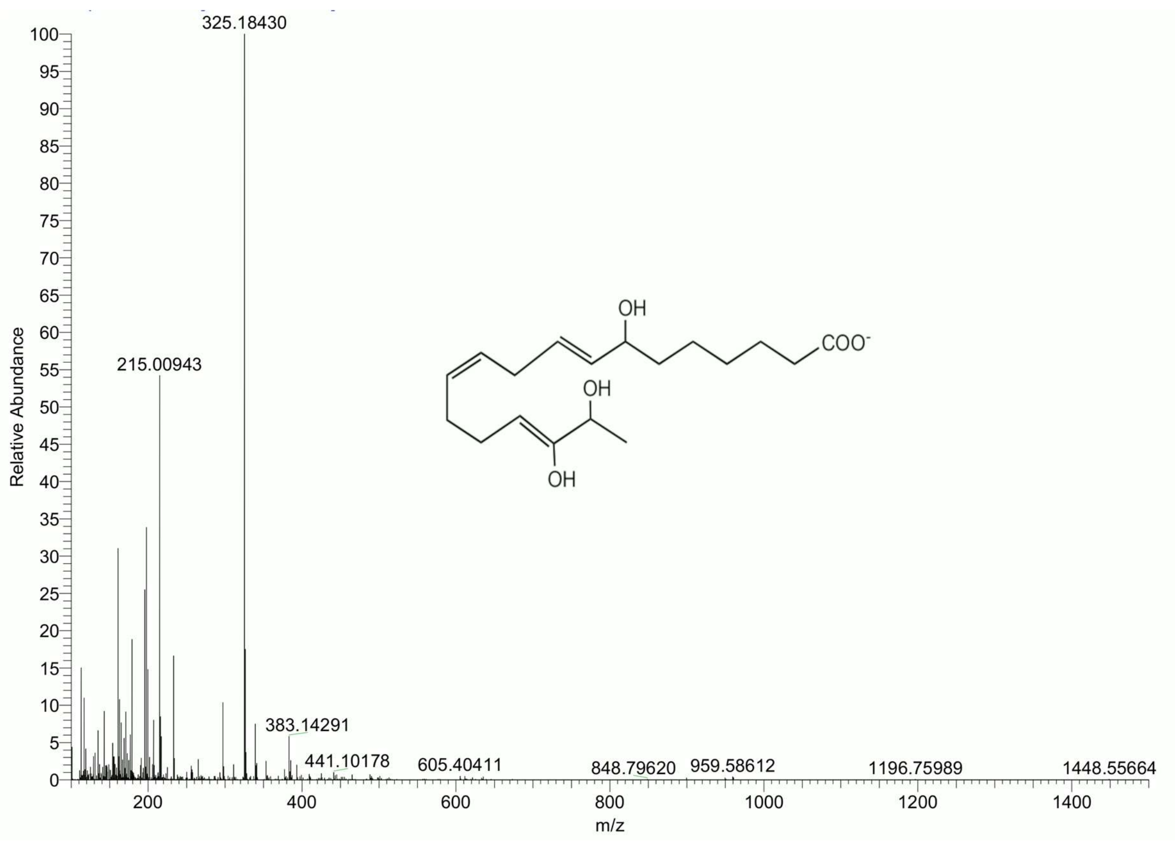

325.18430
215.00943
383.14291
441.10178
605.40411
848.79620
959.58612
1196.75989
1448.55664
Relative Abundance
m/z
OH
COO-
OH
OH

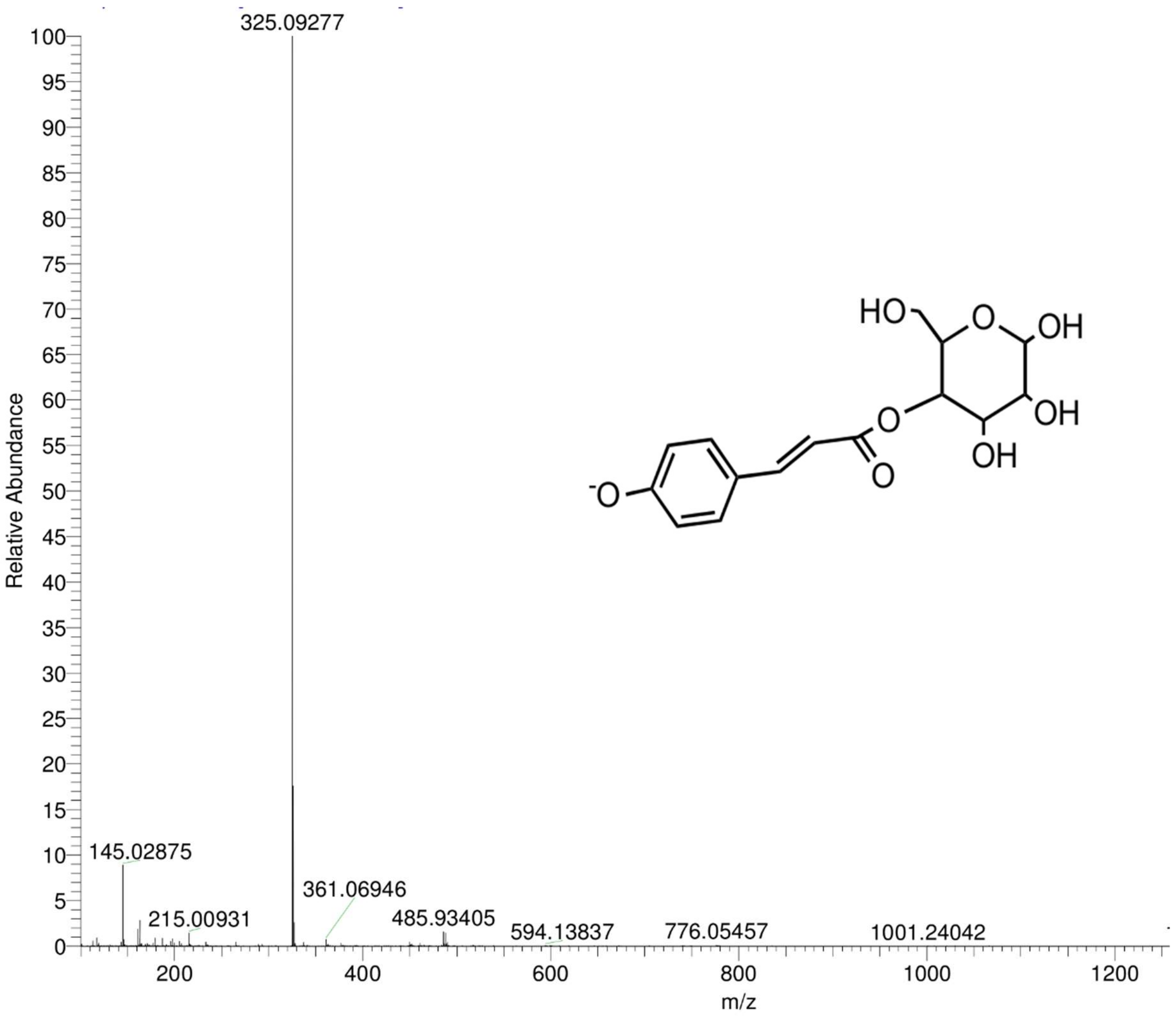
325.09277
145.02875
215.00931
361.06946
485.93405
594.13837
776.05457
1001.24042
Relative Abundance
m/z
HO
O
OH
OH
OH
O
O
-O

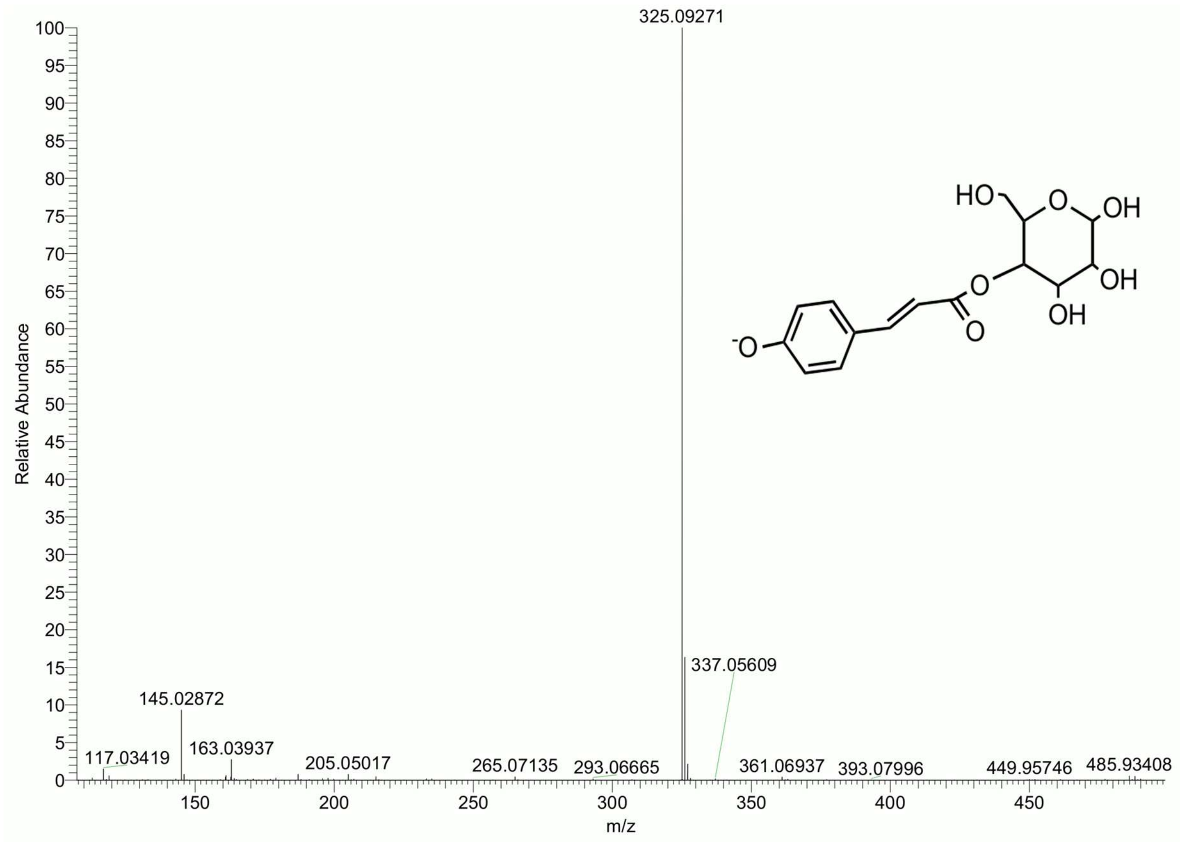

325.09271
337.05609
145.02872
117.03419
163.03937
205.05017
265.07135
293.06665
361.06937
393.07996
449.95746
485.93408
Relative Abundance
m/z
HO
O
OH
OH
OH
-O
O
O

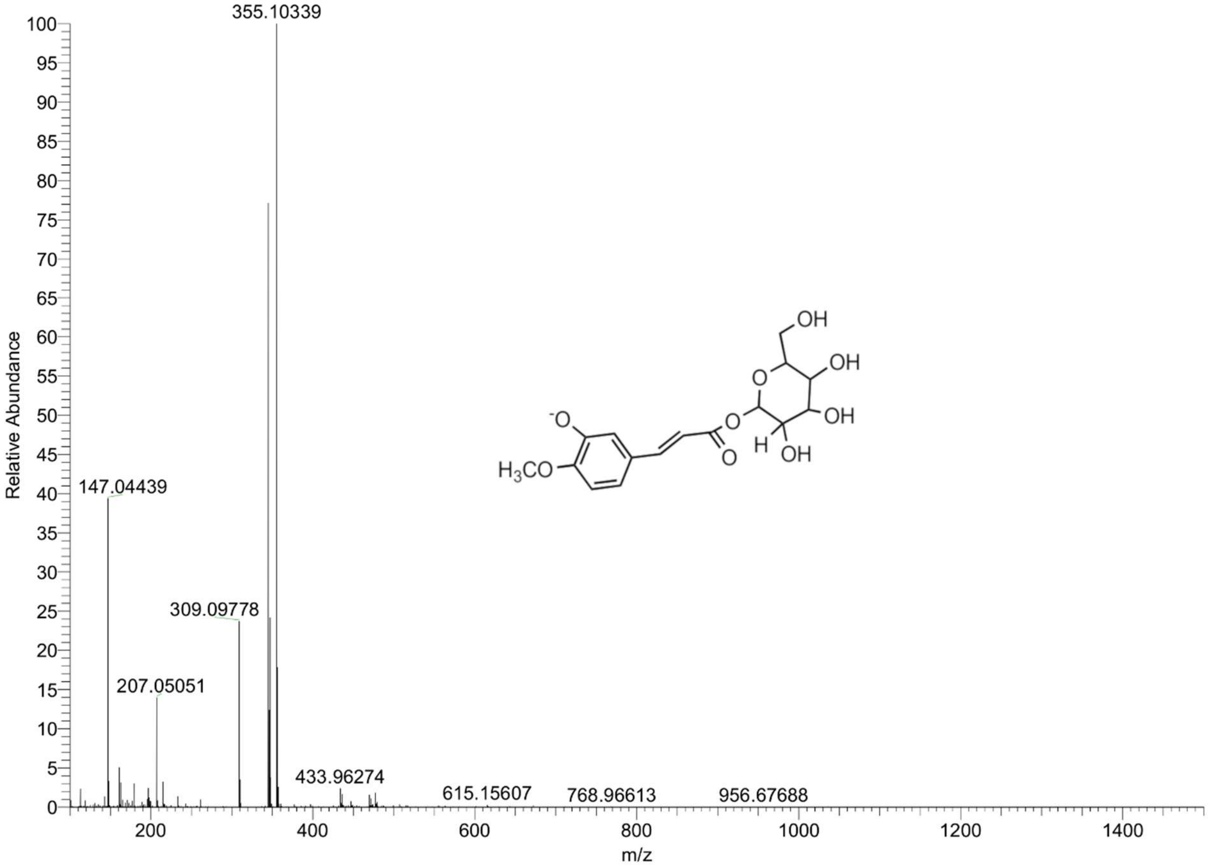
355.10339
147.04439
309.09778
207.05051
433.96274
615.15607
768.96613
956.67688
Relative Abundance
m/z
H3CO
OH
O
H

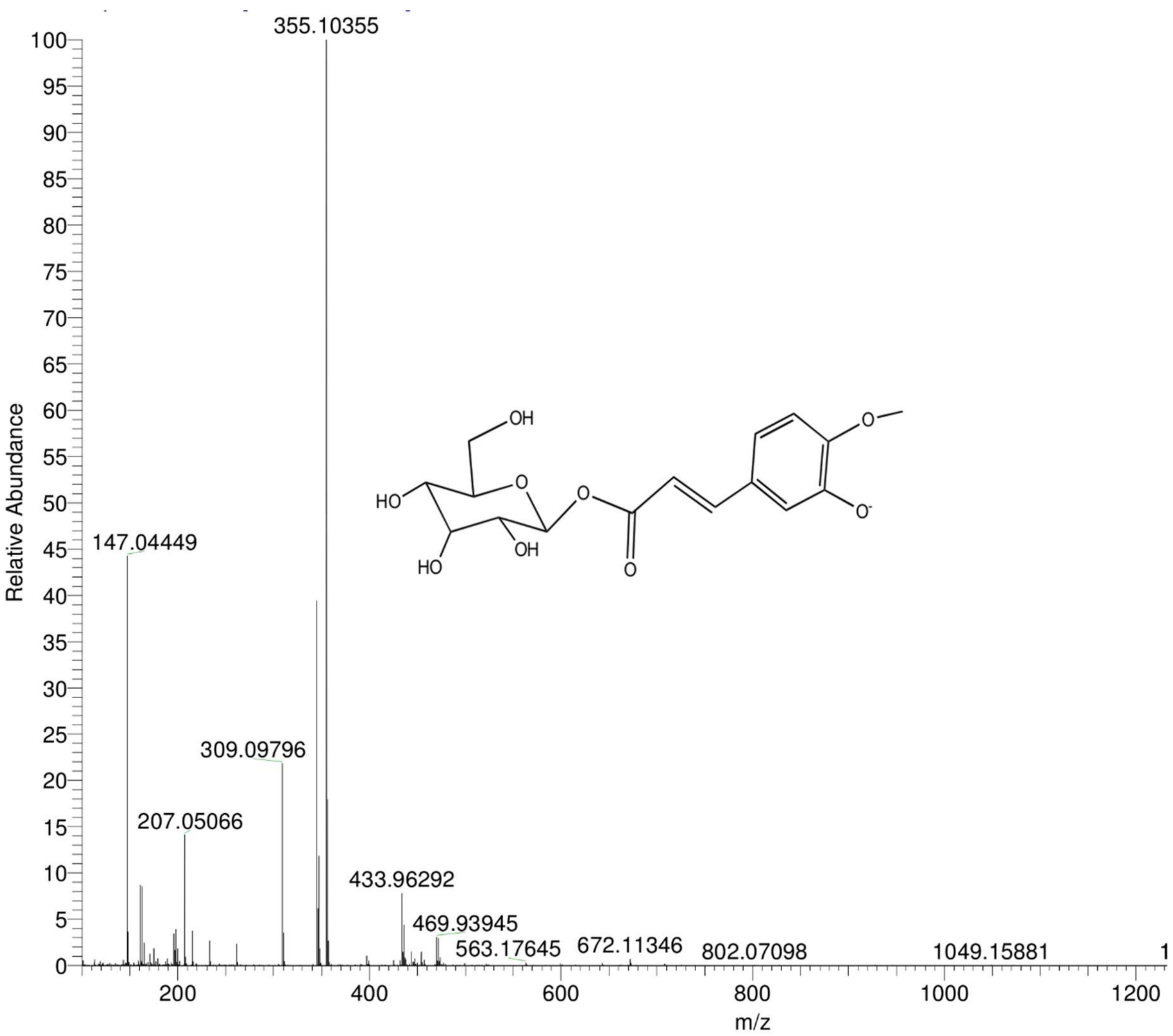

355.10355
147.04449
309.09796
207.05066
433.96292
469.93945
563.17645
672.11346
802.07098
1049.15881
1
Relative Abundance
m/z
OH
HO
O
O
OH
HO
O
O⁻
O

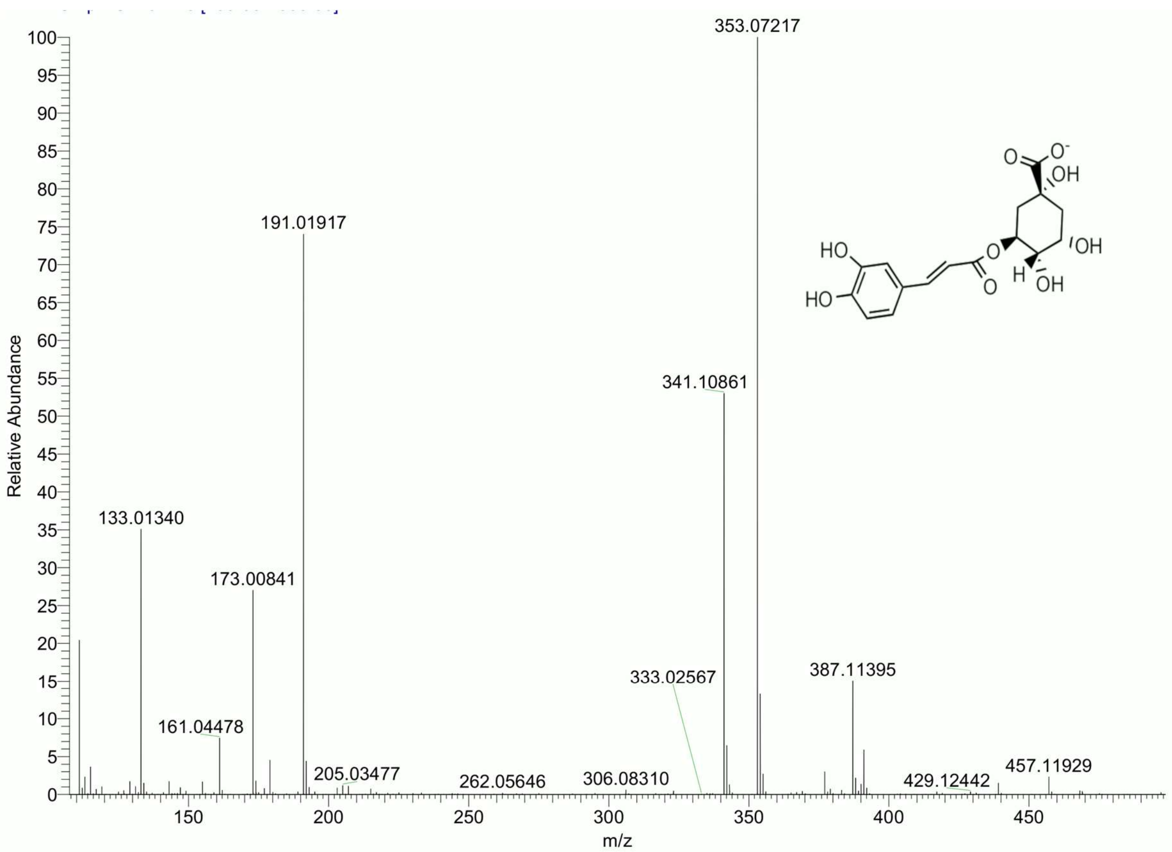
353.07217
191.01917
341.10861
133.01340
173.00841
161.04478
333.02567
387.11395
205.03477
262.05646
306.08310
429.12442
457.11929
Relative Abundance
m/z
HO
HO
O
O
OH
OH
OH
H

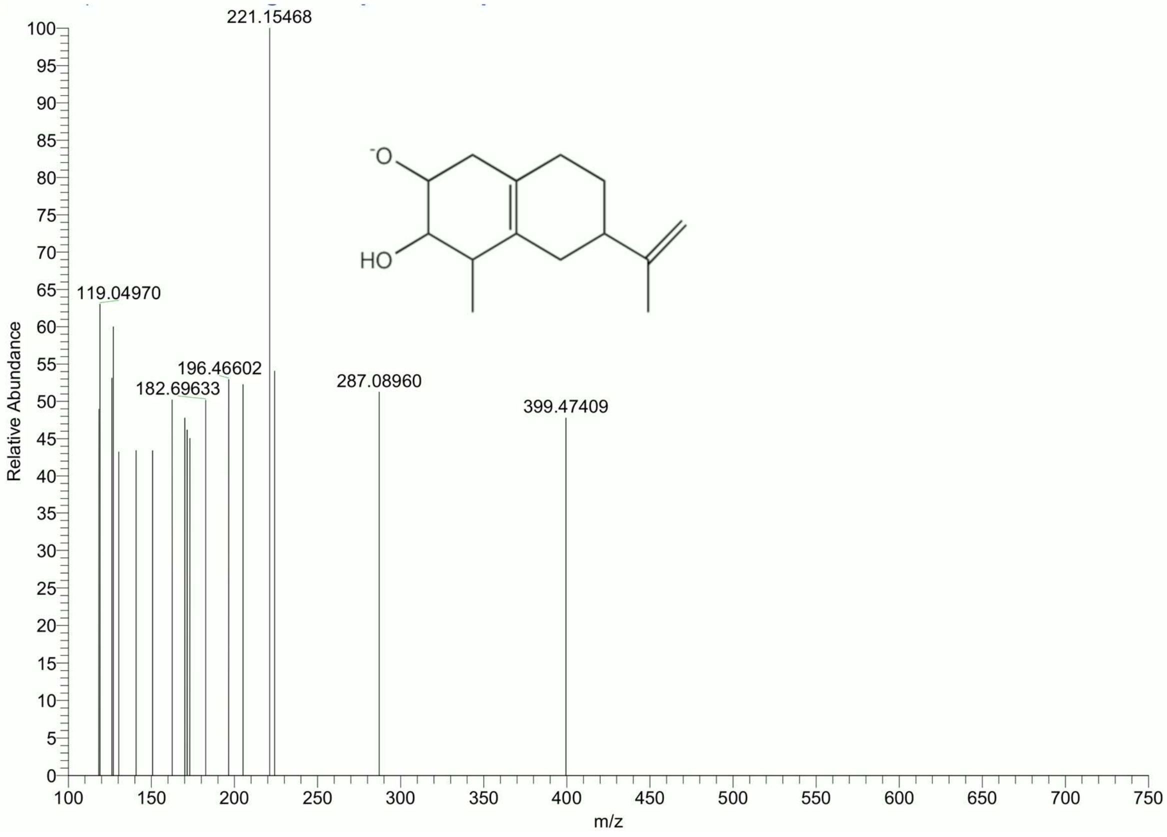
221.15468
119.04970
196.46602
182.69633
287.08960
399.47409
Relative Abundance
m/z
HO

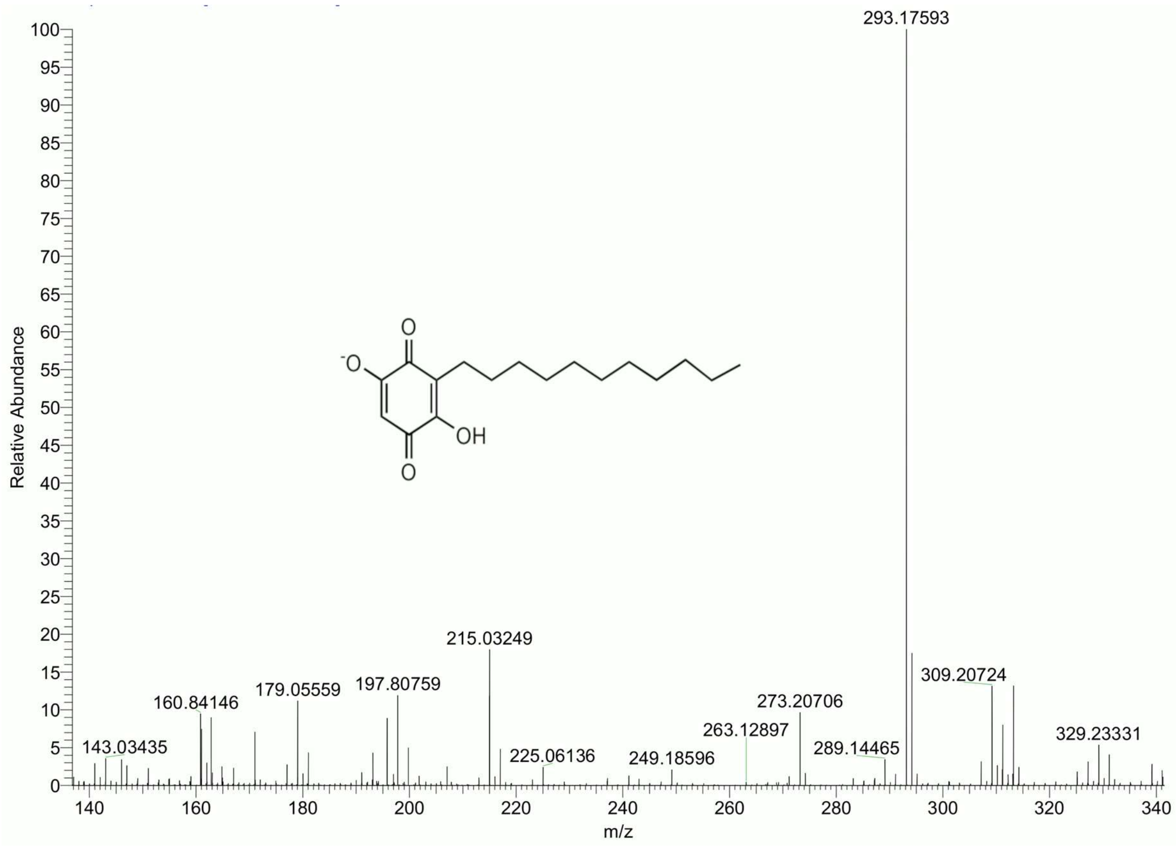

293.17593
215.03249
309.20724
179.05559
197.80759
160.84146
273.20706
263.12897
329.23331
143.03435
289.14465
225.06136
249.18596
Relative Abundance
m/z
O
⁻O
OH
O

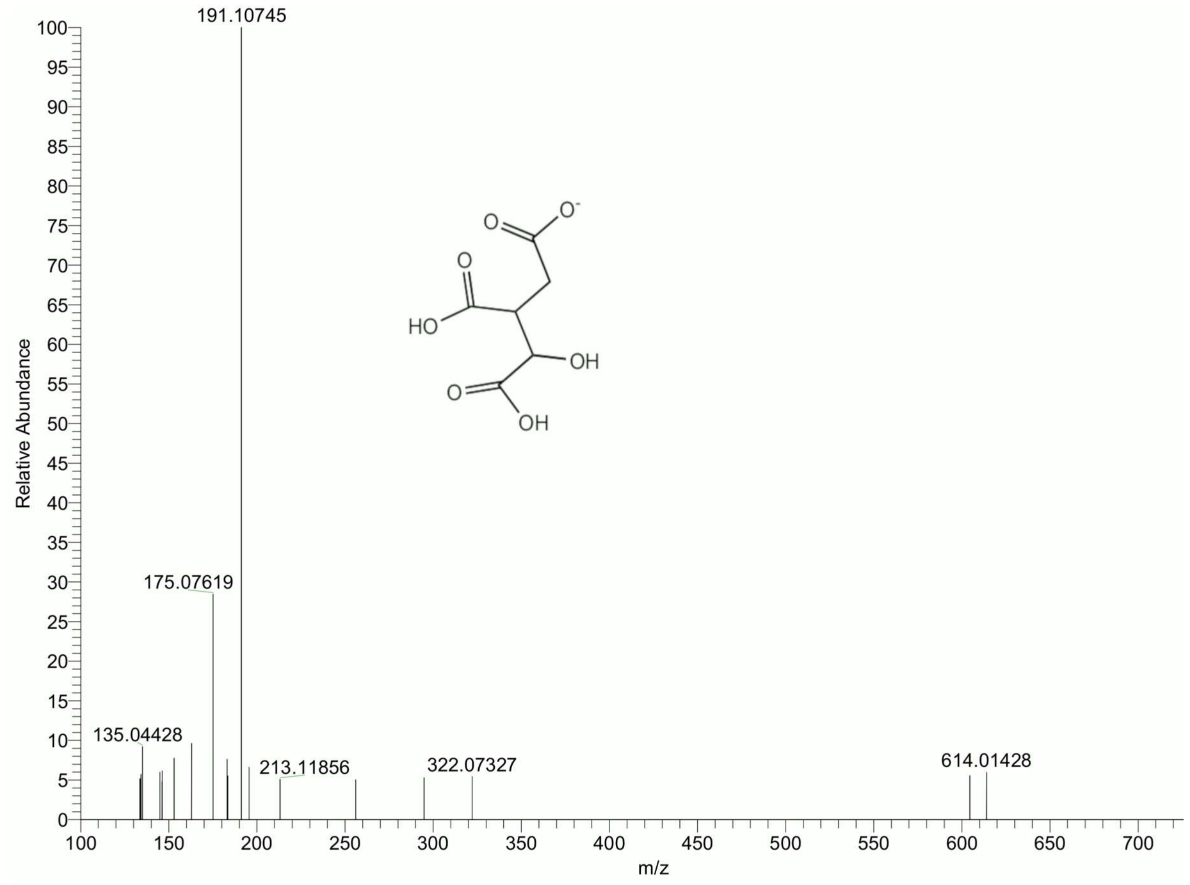

191.10745
175.07619
135.04428
213.11856
322.07327
614.01428
Relative Abundance
m/z
O
O-
O
HO
OH
O
OH

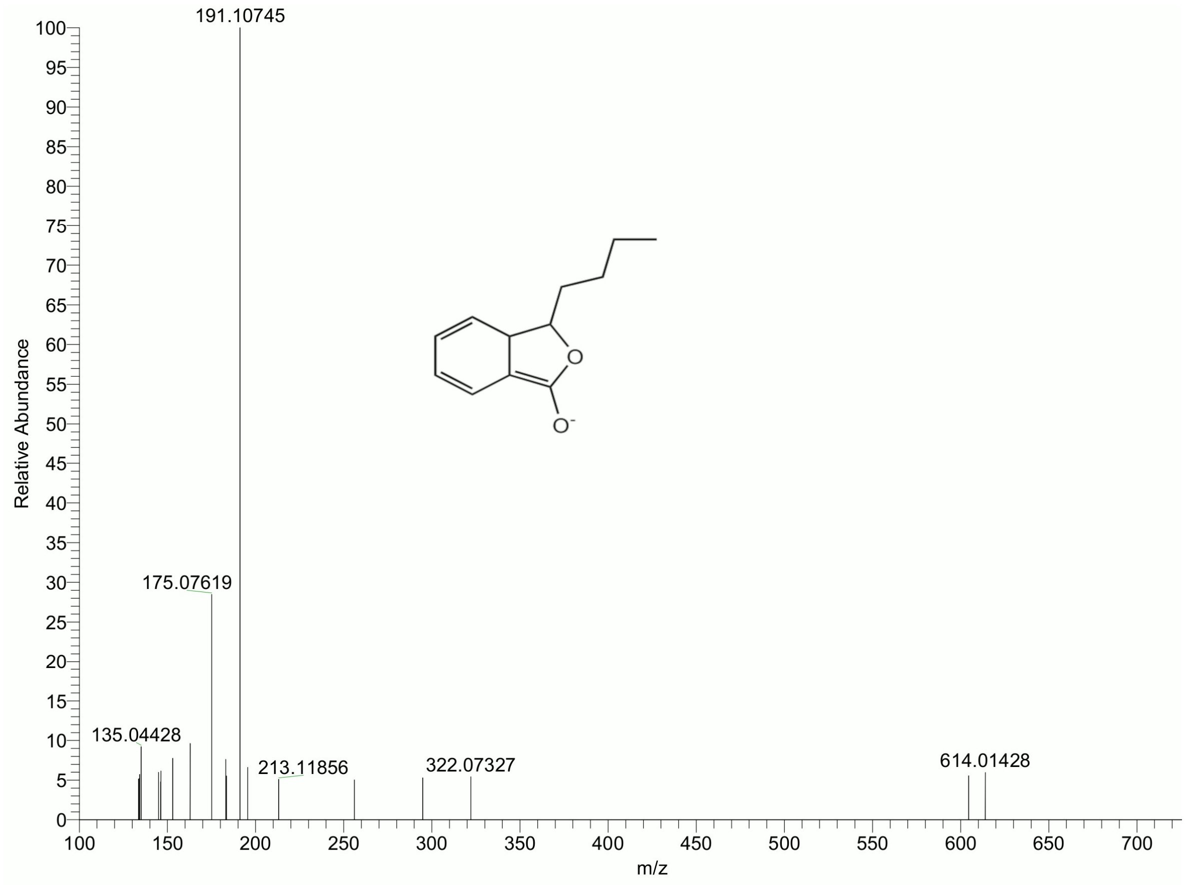

191.10745
175.07619
135.04428
213.11856
322.07327
614.01428
Relative Abundance
m/z
O
O-

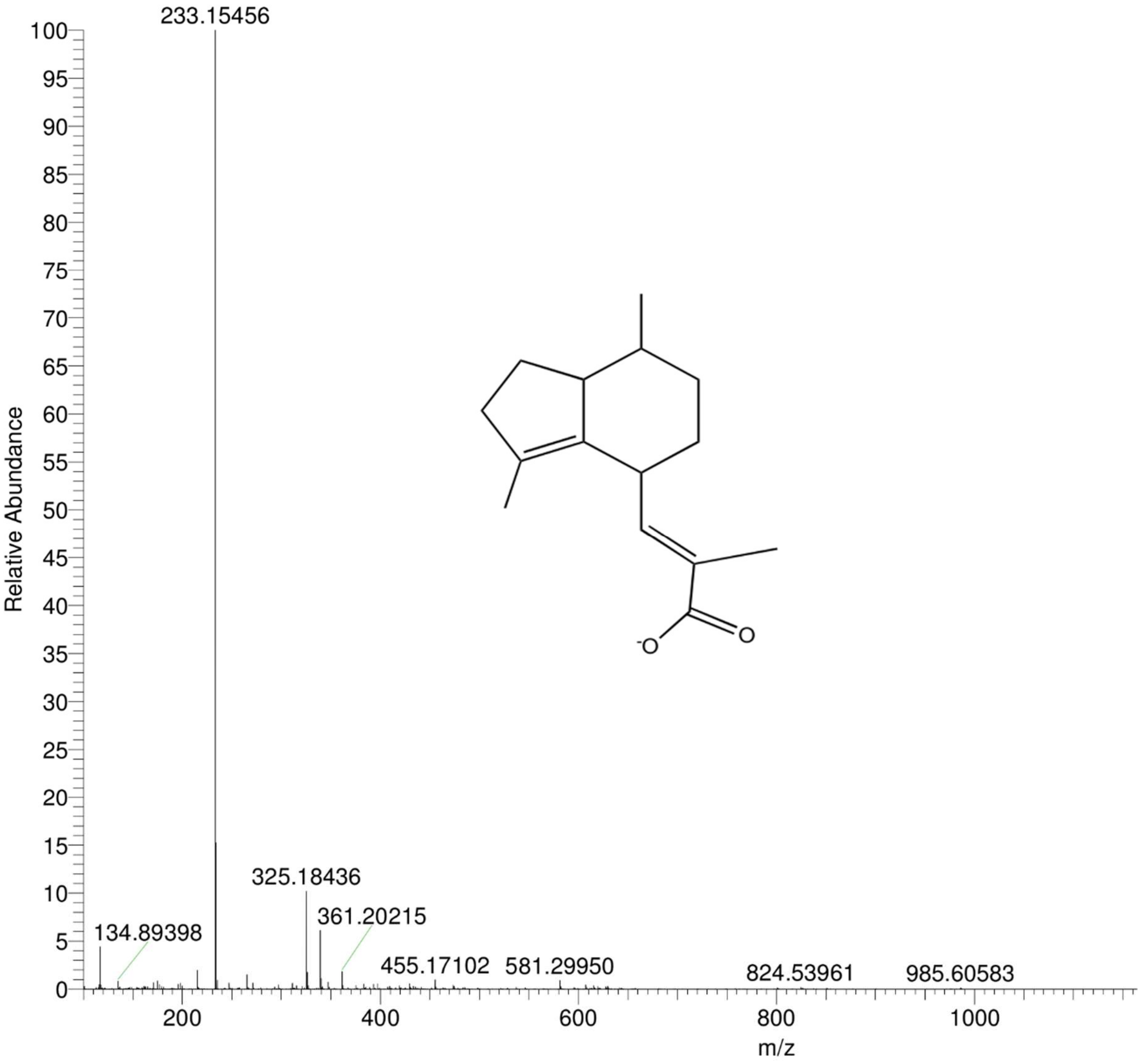
233.15456
325.18436
361.20215
134.89398
455.17102
581.29950
824.53961
985.60583
Relative Abundance
m/z
0
5
10
15
20
25
30
35
40
45
50
55
60
65
70
75
80
85
90
95
100
200
400
600
800
1000
-O
O

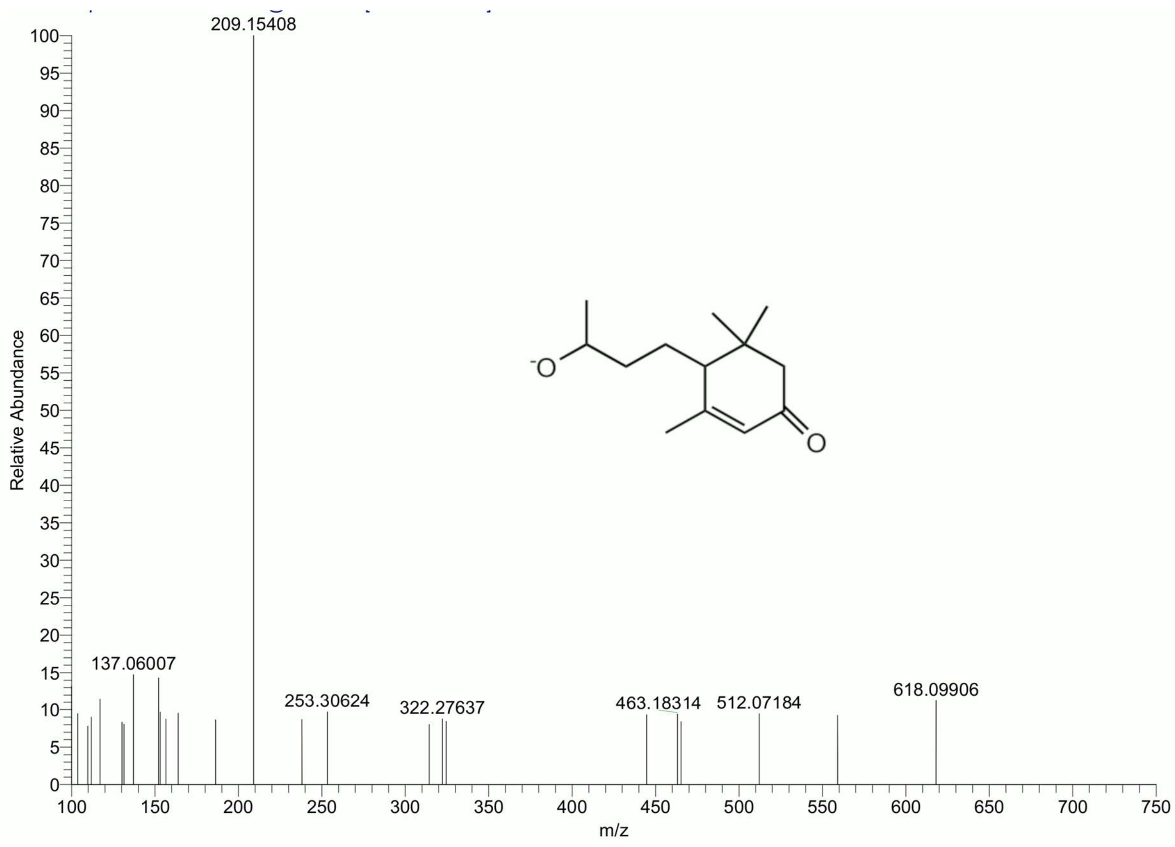
209.15408
137.06007
253.30624
322.27637
463.18314
512.07184
618.09906
Relative Abundance
m/z
O
-O

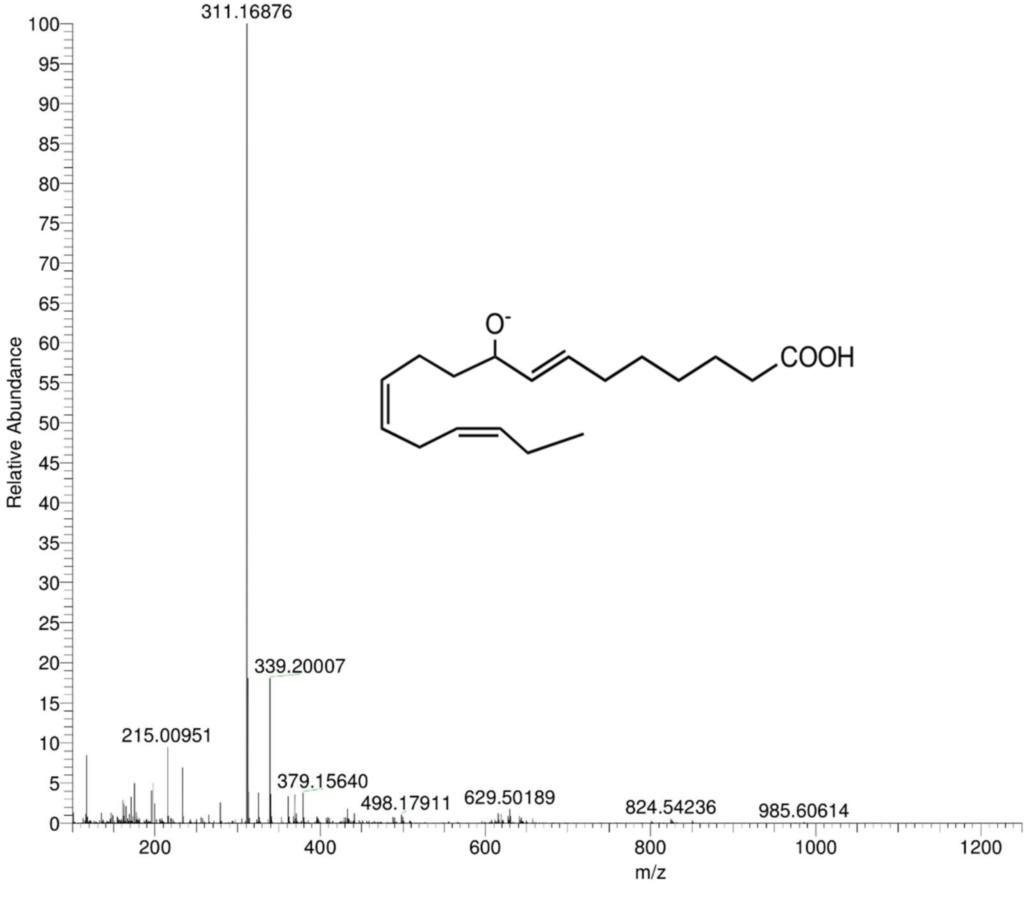
311.16876
339.20007
215.00951
379.15640
498.17911
629.50189
824.54236
985.60614
Relative Abundance
m/z
O-
COOH
